# Elucidating insights on how care was prioritized, adapted, and missed during and post pandemic

**DOI:** 10.1371/journal.pone.0327464

**Published:** 2025-07-17

**Authors:** Lianne Jeffs, Jacqueline Limoges, Tracey DasGupta, Lisa Di Prospero, Alexandra Harris, Jane Merkley, Benjamin Rosen, Adebisi Akande, Frances Bruno, Agnes Black, Linda McGillis Hall

**Affiliations:** 1 Science of Care Institute, Sinai Health, Toronto, Ontario, Canada; 2 Institute of Health Policy Management and Evaluation, University of Toronto, Toronto, Ontario, Canada; 3 Faculty of Health Disciplines, Athabasca University, Athabasca, Alberta, Canada; 4 Interprofessional Practice, Sunnybrook Health Sciences Centre, Toronto, Ontario, Canada; 5 Practice Based Research, Sunnybrook Health Sciences Centre, Toronto, Ontario, Canada; 6 Professional Practice, Unity Health Toronto, Toronto, Ontario, Canada; 7 Executive Office, Sinai Health, Toronto, Ontario, Canada; 8 Department of Psychiatry, University of Toronto, Toronto, Ontario, Canada; 9 Department of Psychiatry, Sinai Health, Toronto, Ontario, Canada; 10 Providence Health Care, Vancouver, British Columbia, Canada; 11 Lawrence Bloomberg Faculty of Nursing, University of Toronto, Ontario, Canada; University of Sharjah College of Health Sciences, UNITED ARAB EMIRATES

## Abstract

Globally, healthcare systems continue to recover and manage system demands, including our sustained HHR pressures exacerbated by the COVID-19 pandemic. Health system leaders need to understand how healthcare was adapted during the pandemic, what contributed to these changes, and the impact of these changes to inform future efforts. The overarching research questions included: What changes to models of care were made during COVID-19 and post-recovery? What factors contributed to changes in models of care? What was the impact of these changes? An exploratory interpretative descriptive qualitative study was undertaken to describe what HHR strategies and changes to models of care delivery were employed during the COVID-19 pandemic and post-pandemic recovery. An inductive thematic analysis was conducted where an investigation team of research staff identified, coded, and categorized prominent themes that emerged in the interview data. A total of 118 participants from a variety of healthcare professionals and leadership positions across five healthcare organizations in the greater Toronto area in Ontario and 1 setting from British Columbia were interviewed. The following three themes were identified during the inductive analysis: 1) prioritizing care based on system capacity, patient volume and complexity; 2) adapting care by innovating, clustering, and taking shortcuts; and 3) being impacted by prioritized and adapted care. Adapting and prioritizing care resulted in missed or delayed care and moral distress in healthcare professionals. Study findings call for leaders to develop and deploy anticipatory adaptive strategies at the organizational level to mitigate pressures related to system capacity and patient volume and complexity. In turn, anticipatory adaptive strategies can guide efforts by healthcare professionals to manage and adapt their clinical tasks, workload, and demands, ensuring patient safety and workforce resilience at the clinical microsystem level.

## Introduction

The novelty of the SARS-CoV-2 virus that caused COVID-19 generated uncertainty and stress related to modes of transmission and high morbidity and mortality rates [1–3]. There were significant disruptions in healthcare delivery from the accelerated cadence and need to reshape and develop new ways of providing health services during and post pandemic [1–5]. Efforts to ‘flatten the curve’ and ensure safe staffing amid continuously changing policy directions required re-organization of resources (e.g., personal protective equipment (PPE), vaccines) and re-imagining of health human resources (HHR) (e.g., team-based models of care, re-deployment) [4–8]. Underpinning these efforts were the setting of priorities and allocating resources – albeit scarce at times – which were under great scrutiny from the tension between increased demand for services and widening HHR shortages [7]. Leaders had to make and continue making decisions and ‘trade-offs’ across several levels – system (macro); organizational (meso); and clinical interactions (micro) on which patients receive (or should receive) services and how quickly they were treated [7]. To date, the majority of empirical knowledge around the decision-making choices focuses on physical distancing, minimizing travel, mask wearing, lock downs, vaccinations [6], and changes to workflow [4].

Globally, healthcare systems continue to recover and manage system pressures and demands, including our sustained HHR pressures exacerbated by the COVID-19 pandemic [1]. There is a small body of literature that focuses on how care was adapted to meet these changing demands. For example, nurses reported clustering their care by grouping care activities together during the early phases of the pandemic to preserve PPE [7,9], minimizing time spent in the patient rooms [10], and being more efficient [11]. Nurses also reported prioritizing nursing care tasks during the COVID-19 pandemic (e.g., planning and communication over nursing care tasks related to the clinical domain) [12]. However, less is known around the decision making and priority setting that guided care and processes of care at the clinical microsystem (e.g., where patients and health care providers interact) [13] during the pandemic and post-pandemic recovery. This knowledge gap is important to address as the health system continues to face challenges post pandemic. Health system leaders need to understand how healthcare was adapted during the pandemic, which changes were improvements that are sustainable, and which must be developed. As such, a study was undertaken to explore the following research questions: What changes to providing care were made during COVID-19 and post-pandemic recovery? What factors contributed to changes to providing care during COVID-19 and post-pandemic recovery? What was the impact of the changes to providing care during COVID-19 and post-pandemic recovery? This paper provides key findings from this study that describes how care was prioritized and adapted, resulting in missed care that impacted patients, care partners, and healthcare professionals at the clinical microsystem level.

## Methods

### Study setting

Healthcare professionals were recruited from 6 acute care hospitals (critical care, inpatient, and outpatient areas/units in hospitals), complex care, rehabilitation care, and long-term care settings. Five hospitals were academic teaching hospitals and one was a large community-based hospital.

### Study design

An exploratory interpretative descriptive qualitative study [14] was undertaken to describe what HHR strategies and changes to models of care delivery were employed during the COVID-19 pandemic and post-pandemic recovery. Interviews were conducted in six hospitals in two different provinces of Canada over the course of close to a year [December 7, 2022 to October 19, 2023].

Full Ethics Statement: Research Ethics Board (REB) approvals were obtained from all participating sites and are outlined in Table 1 with approval letters included in S1 File. Written and verbal consent were obtained from all participants by a research assistant prior to interviewing.

**Table 1:** Study sites research ethics board numbers.

Site	Research Ethics Board Approval Number	Interview Dates
Providence Health REB	H22-02792	02/22/2023 − 06/07/2023
University Health Network REB	23-5214	08/21/2023 − 10/12/2023
Sunnybrook Health Science Centre REB	5571	04/12/2023–06/28/2023
Sinai Health REB	22-0153-E	12/07/2022–06/29/2023
Scarborough Health Network REB	MIS-23–006	06/28/2023–10/19/2023
Unity Health Toronto REB	23-058	08/31/2023–09/07/2023

### Recruitment and data collection methods

A purposeful, snowball sampling strategy was employed to recruit study participants who had experienced a change in models of care and/or staffing (e.g., redeployment) and leaders (e.g., unit managers, team leaders, etc.) who were involved in redeploying and educating/training staff were included in this study. Research staff received training on how to conduct scientific interviews from a doctoral prepared qualitative researcher and participated in mock interviews prior to conducting the interviews. The interviews were conducted by 6 research staff virtually using a one- semi-structured interview guide (approximately 60 minutes in length) with study participants. See S2 File. Participants were asked about their experiences and perceptions associated with changes to models of care and staffing. Interviews were recorded and transcribed verbatim by a research staff or by a professional transcription service with a research staff verifying the transcription with the original digital recording.

### Data analysis

An inductive thematic analysis [15,16] was employed involving an investigation team of four research staff who identified, coded, and categorized prominent themes that emerged in the interview data. Specifically, the analytical process involved an independent line-by-line review of all transcripts by two individuals of the research team to identify sections of text that served as codes, which were then rolled up to categories. A coding schema and codebook were developed through consensus and discussed iteratively by the research team throughout the analysis process to ensure consistency across coders and accuracy of the code descriptions [15,16]. NVivo software [NVP20-JZ000-IH020-YR08T-M51UW, NVP20-JZ000-IH020-YR08U-M7U7Q, and NVP20-JZ000-IH020-YR08V-0POOW] was used to store data and enable cross comparison within the narrative dataset. As a final step, to ensure saturation and methodological rigor, the principal investigator (LJ) reviewed the emerging and final coding schema with the original transcripts [17].

### Sample characteristics

A total of 118 participants from five healthcare organizations in the greater Toronto area in Ontario and one setting in British Columbia were interviewed. Table 2 provides an overview of the demographics of study participants.

**Table 2:** Study demographics.

Demographic Variable	Number of Responses	%	Category
Age	59 48 11	49% 41% 10%	41-60 age range 20-40 age range 60 age range
Gender	106 12	90% 10%	Female Male
Ethnicity	48 24 20 11 4 3 8	41% 20% 17% 9% 3% 3% 7%	Caucasian/White Asian Canadian Mixed Race European No Response Other
Profession/Position	49	42%	Health Disciplines
	46	39%	Nurses
	12	10%	Leadership
	4	3%	Physician
	7	6%	Other

## Findings

The following three themes were identified during the inductive analysis: 1) prioritizing care based on system capacity, patient volume and complexity; 2) adapting care by innovating, clustering, and taking shortcuts; and 3) being impacted by prioritized and adapted care. Table 3 provides theme and sub-theme definitions. S3 File provides the dataset.

**Table 3:** Themes and sub-themes definitions.

Theme Definitions	Definitions	Sub-Theme Definitions
Theme 1: Prioritizing Care Based on System Capacity, Patient Volume and Complexity	This theme captures the interplay and influence of contextual factors (system capacity, patient volume and complexity) that necessitated the prioritization of care amid the COVID-19 pandemic and post pandemic recovery. Prioritizing care reflects the decisions involved in determining what care was to be provided and changes to care including being omitted, reduced, rationed, and/or delayed.	Sub-Theme 1.1: System Capacity reflects the contextual factor of how the severe HHR shortages impacted the ability to provide routine and standard of care.
		Sub-Theme 1.2: Patient Volume and Complexity reflects the contextual factors of increased patient volume and complexity (urgency, acuity, health care needs) impacted the ability to provide routine and standard of care.
Theme 2: Adapting Care by Innovating, Clustering, and Taking Shortcuts	This theme captures how participants described adapting what and how they provided care amid the pandemic and HHR shortages.	Sub-Theme 2.1: Innovating reflects the adaptions in providing care that were new and innovative.
		Sub-Theme 2.2: Clustering reflects the adaptations in providing care by clustering and bundling care during the same clinical interaction.
		Sub-Theme 2.3: Taking Shortcuts reflects the adaptations in providing the most necessary care or bare minimum.
Being Impacted by Prioritized and Adapted Care	This theme captures the impact of the changes and adaptations to providing care on patients (leaving care undone/missed care) and providers (moral distress).	Sub-Theme 3.1: Leaving Care Undone/ Missed Care reflects the adaptations in providing care including care that was not done or delayed and the negative consequences experienced by patients and care partners.
		Sub-Theme 3.2: Experiencing Moral Distress as a Result of Impact on Patient Care reflects healthcare providers’ experiences associated with leaving care undone/missed care.

### Prioritizing care based on system capacity, patient volume and complexity

This theme reflects contextual factors including system capacity and patient volume and complexity that necessitated the prioritization of care amid the COVID-19 pandemic and post-recovery. Prioritizing care involved decisions related to what care was to be provided and changes to care including what would be omitted, reduced, rationed, and/or delayed.

1. **System capacity.** Participants described working short staffed to mitigate the absenteeism from staff who were infected with the COVID-19 virus and/or had to take a leave of absence due to challenges with mental health. HHR was further impacted by staff leaving healthcare organizations, staff having limited capacity to work shifts above their usual hours, and difficulties recruiting new healthcare professionals. These severe staffing challenges had immediate implications for care delivery, affecting the provision of routine, standard care and reducing the amount of time a healthcare professional could spend with a patient. Efforts to curb disease transmission led to the cancellation and slowing down of healthcare services. This was highly pronounced among surgical units, as pandemic restrictions halted elective surgeries and non-urgent procedures. Participants also described pivoting to virtual care or no longer being able to provide care in the home setting. The following narratives depict this theme:

*“It’s an ongoing concern that we’re struggling with recruitment to fill nursing positions. I can see and feel the difference in the department when we’re operating short on nurses. Types of care have shifted because we just can’t do that routine thing anymore…We routinely have to close care spaces because we don’t have sufficient staffing.”* [Social Worker, Site 1]

“*There were times that you’re in one room for a long time and you can hear the call bell of your other patient but no one is able to go into that room. So you’re rushing in and out..*” [Registered Nurse, Site 2]

*“I went from 100 percent in person work type to overnight shutting down our clinic. We had to pivot over night over the weekend to virtual care.*” [Dietitian, Site 1]

2 **Patient volume and complexity.** Participants also identified how patient volume (e.g., increased numbers of patients) and patient complexity (urgency, acuity, health care needs) led to considerable changes in the way care was delivered (e.g., change in protocols, practice). Participants shared that patients who were more symptomatic, sickest, and with higher care needs were prioritized to receive care. As one participant noted “*I would prioritize by seeing the most sick patients”* [Respiratory Therapist, Site 3] while others shared they would focus on the highest needs (e.g., airway, breathing, circulation, medications) and *“not doing all the extras”*. Trying to focus on basic, fundamental care needs (e.g., hygiene and counselling) was also described, as one participant noted *“prioritize the skin, skincare, mouthcare, we still tried to make sure the patient looked nice as a human”* [Registered Practical Nurse, Site 4]. Participants described the uncertainty and conflicting knowledge on how best to provide care to COVID-19 patients (e.g., proning patient by placing on stomach and physical distancing) and how this impacted how care was provided. The following narrative provides examples of this sub-theme.

*“The most symptomatic patients were prioritized and seen first and managed more acutely. Those that were more stable may have not gotten a check-in on a daily basis.”* [Advanced Practice Nurse, Site 3]

*“You go into that mode when you know the need. Prioritize to see which patients needs to see at what time and then together we work together to see the patient and meeting the needs of the patients during the pandemic.”* [Clinical Nurse Specialist, Site 2]

*“We have so many unknowns and if the rules are changed, especially in terms of isolation was changing on a daily basis, we’re learning on the go how this virus behaved and what was happening.”* [Physiotherapist, Site 2]

Some participants (mainly in Ontario) shared that the Provisions of Care Guidance Documents developed provincially (Ontario Health) and regionally (Toronto Region Hospital Operating Table) were used organizationally. These documents were used to guide decisions and were adapted into tools (e.g., local practice alerts, provisions of care documents) around prioritizing care in clinical areas and by professional disciplines (e.g., pharmacy and nursing). As noted in the following narratives:

*“We made a list of things that were first priority, second priority, third priority. It was a day-to-day discussion. The provisions of care [decision-making guideline] was more a reaction or a strategy based off of the pressures of the system and a way to mitigate risk and continue to provide care.”* [Patient Care Manager, Site 4]

*“Having that [guidance documents] as a tool is value add for going forward. We’re moving into provisions of care and there was focusing on fundamentals of care, pain management, communication to families. We listed the things that were higher priority and then encouraged more targeted interventions and documentation. We were able to say, “we’re going to go into provisions of care for two weeks, or for one week.”* [Director, Site 4]

### Adapting care by innovating, clustering, and taking shortcuts

This theme captures how participants described adapting what and how they provided care amid the pandemic and HHR shortages.

1 **Innovating.** Participants described how care was adapted in daily practice as *“trial and error”*; making *“wild adaptations”,* making *“it up in the moment”; “things on the fly”*; and thinking *“outside the box”*. Participants provided examples of adaptations and innovative practices including tailoring provisions of care guidance documents to local contexts; a decision-making algorithm in pharmacy; proning patients in ICUs; piggybacking infusion medications; monitoring patients diagnosed with COVID using binoculars, having IV poles outside their rooms, and relieving stress by having IPAC-safe toys and individual activity packages with mindful colouring. The following quotes provides examples of this theme of adapting through innovating.

*“We got some input from the pharmacist and we developed the top priority things you’ve got to do, We helped developed an algorithm when you’re really busy, here is what you should focus on and don’t worry about things you would have done for sure.”* [Pharmacist, Site 4]

*“Nurses being innovative with ways of making sure we didn’t have to go in the rooms as much and piggybacking infusion medications.”* [Registered Nurse, Site 1]

*“Everyone’s ability to adapt and do what was needed for the patient was amazing and times we would be proning six patients in the ICU and people just did what they had to do.”* [Respiratory Therapist, Site 3]

*“We had to think outside the box in problem solving. We have an activity box on the unit for colouring or word searches or mindfulness colouring, and stress relieving IPAC-safe toys that can be viroxed, stress balls. We would give them an activity package to help them [patients] pass time.”* [Social Worker, Site 4]

2 **Clustering.** Participants also provided examples of clustering and bundling care including assessing, turning, bathing, feeding, and administering medications to patients during the same clinical interaction. The clustering of care for patients with COVID at the end of their shift was recommended as a strategy to prevent the spread of the virus. Participants also shared that clustering and bundling care was necessary to limit interactions due the pandemic restrictions and was the most efficient way to provide care. Underpinning clustering and bundling care was the need to be *‘thoughtful’* and *‘mindful’* of the time spent with patients to do what was needed to be done. As described by one participant *“We tried to bundle our care as much as we could, that was just something that was sort of easier for us to do, prioritizing really what they needed”* [Nurse Practitioner, Site 3]. The lack of family members and support people to provide care was identified as a contributing factor for the need to cluster care. The following narratives also illustrate this theme.

*“At one point patient care was clustered so you minimized the amount of time that one would enter a patient’s room - whether it be their hygiene care, feeding the patient, repositioning the patient.”* [Clinical Nurse Specialist, Site 2]

*“If I had COVID-positive patients, I would leave those to the end of my rounds. I would be thoughtful about what I needed to order and limit the interactions.”* [Nurse Practitioner, Site 3]

*“I’ve learned to bundle my care a lot more in the pandemic because there was a lack of people who are support people for my patients – family, support personnel, other peopleI had to really be mindful of my time in which I spent with a patient.”* [Physiotherapist, Site 3]

3 **Taking shortcuts.** Participants also described taking shortcuts and providing a*“bare minimum”* of care by doing what was necessary for patient care, safety and well-being. As one participant noted *“we streamlined and changed the provisions of care of what our bare minimum would be when we are at low staff or when the surge is too high.”* [Pharmacist, Site 4]. Nurses were the predominant healthcare professional who reported taking shortcuts. One clinical nurse specialist shared the need to “*do exactly what you need to do to keep the patient safe and follow their care regimes or their orders to the best of your abilities. At minimum you’re crossing your fundamentals”* [Clinical Nurse Specialist, Site 4]. Taking shortcuts included streamlining and reducing the type and frequency of care provided or not providing care at all (e.g., bathing, emotional support, turning). Other narrative excerpts from nurses include:

*“If you didn’t do these shortcuts you wouldn’t do everything that you needed in that shift, that were necessary for patient care. For example, you were giving a patient medications – they can’t swallow the medications, policy would be you would crush each medication separately and administer them one by one, but that could take a real long time, so we’d like crush all of them and put them in one cup and then give it all at once.”* [Registered Nurse, Site 1]

*“We tried to wash them thoroughly but during COVID because you have extra things to do and responsibility, you only wash whatever is important. Today I washed the patient thoroughly, and the second day I did not because I have to do procedures that are more important than washing the patient.”* [Registered Practical Nurse, Site 4]

*“The shortcut would be that you weren’t doing the emotional support that would typically happen when you’re fully staffed and you have more time. You were doing very much the physical aspect, the check-in, making sure the symptoms were well managed. But you weren’t necessarily spending time being present, bearing witness and providing that emotional component”* [Advanced Practice Nurse, Site 3].

### Being impacted by prioritized and adapted care

This theme elucidates the impact of the changes and adaptations to providing care on patients (leaving care undone/missed care) and providers (moral distress).

1 **Leaving Care Undone/Missed Care.** Instances of care left undone or missed care were also attributed to the cancelling and slowing down of services. The most commonly shared missed care included washing and hygiene, relational care, turning, mobilizing, and therapy. Participants stated that they did not have time to do what they viewed as *“extra”* such as washing a patient’s face or providing physical therapy and not being able to attend to their patients’ basic needs including therapeutic conversations and interactions. As one participant described the lack of time to intervene also put patients at risk for pressure injury “*If we’re limiting the amount of time we’re spending with patients we don’t get frequent intervention then there is the opportunity for pressure injury”* [Clinical Nurse Specialist, Site 2]. The focus on addressing immediate and priority care needs as a mitigating strategy to manage the high patient volume and complexity amid HHR issues and restrictions resulted in missing the relational and therapeutic aspects of care. In some situations, care was delayed (e.g., medications and procedures done late). This theme is illustrated in the following quotes:

*“There wasn’t time to do any of the additional relational things with patients like sitting and talking to them for a while. We tried to do but there wasn’t enough time nor were there enough resources. We were operating in crisis mode at the height of the pandemic.”* [Director, Site 2]

*“Once we started having those conversations about lessening your visits to the room we did notice a peak in pressure ulcers. We did notice a peak in patients falling. We did notice that nurses were not necessarily communicating with family members as much.”* [Clinical Practice Leader, Site 5]

*“When you then bundle your care and only go in every few hours and the isolation that people experienced often resulted in hypo activity and depression and all of that impacts the way we respond to illness and injury.”* [Clinical Nurse Specialist, Site 2]

#### Experiencing moral distress as a result of impact on patient care.

Several participants acknowledged the negative consequences that patients and care partners experienced due to missed care and the inability of the healthcare professional to provide holistic care. Participants also described experiencing moral distress when they were not able to provide a level of care patients needed. For some participants, they described this is a *“background movement”* that has been normalized into current practice. The following narratives provide examples of this theme.

*“The basic nursing practice has fallen to the wayside. Overall, there’s less connectivity, there’s also been a huge backwards movement regarding patient and family centred care. Now we’re at the place where nurses think that it’s normal for families to only be allowed at the bedside for a limited amount of time.”* [Registered Nurse, Site 1]

*“We changed our provision of care, we’re used to providing a certain standard of care and going below that standard was a hard adjustment. I am having a hard time going back to my previous standard of care. I am operating at the low of what I used to do just because of the increased workload and the higher turnover.”* [Pharmacist, Site 4]

*“Basic care was missed and the nurses would be morally distressed and they feared that patients are not getting better because there’s no complete care being provided. I’m trying to turn them but there’s no time for that.*” [Registered Nurse, Site 1]

## Discussion

To our knowledge, this is the first study to elucidate the decisions and processes related to how care was prioritized and adapted at the clinical microsystem level during the pandemic. Further, this study revealed under-lying decision-making processes that resulted in missed or substandard care. Specifically, our study unpacked how leaders and clinicians prioritized what type and ‘dose’ (level and quantity) of care would be provided (or not) based on the interplay of system capacity (HHR and supply chain), patient volume (numbers of patients requiring care), and complexity of care (both at the individual patient level and overall system level) in a rapidly changing environment. This interplay involved prioritizing the sickest and most complex of patients to meet the highest needs with fundamental care needs being left undone or rationed as evidence on how to provide care evolved over the course of the pandemic. This was echoed in previous research where the urgency of the patient’s condition while ensuring patient safety were the most decisive criterion in care provision triage for healthcare professionals [18–21]. For example, a study in the ICU revealed that healthcare professionals prioritised care within and between acutely ill patients with oral care, wound care and dressing changes being the first to be omitted [20]. In an umbrella review, the gravity and severity of a patient’s health condition and status dictated the urgency and intensity of care required [21]. Further, heightened vigilance and immediate attention was enacted to address patients facing more acute or complex health challenges [21].

Although not specific to the COVID-19 pandemic, similar findings have been reported around nurses prioritizing care in attempts to address the diverse needs and level of severity of patients to the best of their abilities [22,23]. Our findings around stopping services and delaying care adds to the growing literature base on how healthcare organizations made trade-offs with respect to which patients would receive services and how quickly certain patients would be treated [1,4]. For example, as the demands for urgent services significantly increased, some care processes and tasks were deprioritized [1,4]. Stopping or restricting clinical activities, provision of care, supplies and services were identified within a taxonomy of adaptive strategies that leaders deploy to mitigate health system pressures [1]. Our study findings provide empirical support for this taxonomy [1] as leaders and health care professionals were constantly prioritizing and re-prioritizing patients and temporarily stopping or delaying some clinical activities and types of care. Specifically, our findings around using the guidance documents, creating lists, algorithms, and practice alerts to prioritize care are examples of what is referred to as *“anticipatory adaptations”* to manage workload which includes forward planning, contingency planning, creating or adapting protocols in the taxonomy^.^ [1]. The documents used in our study enabled healthcare professionals to be more prepared to make and implement decisions around prioritising and adapting care to meet the evolving healthcare pressures in daily practice.

Our second theme describes how healthcare professionals made adaptations in the moment to providing daily care. This finding is similar to what is referred to in the aforementioned taxonomy as doing *“on-the-day adaptations”* and *‘task shifting”* to manage workload demand and the use of resources in times of increased pressure [1]. Within this conceptualization, teams and individual professionals are constantly adapting making changes to staffing and existing resources, patient flow, and functioning of services in efforts to maintain a reasonable quality and safety of care within the available constraints [1]. In our study, healthcare professionals shared examples of how they provided care in innovative ways amid the pandemic with restricted access to family and caregiver resources (e.g., adapting provision of care guidelines and decision tools, using binoculars to monitor patients). A multi-national case study (Brazil, Canada and Japan) also reported the nature of the COVID-19 pandemic forced the health care system to reshape health services and develop new ways of undertaking innovative changes in service provision, processes, organizational structure, and hospital operational policy [3]. Specifically, the authors reported the development and adaptation of standard operating procedures, implementation guides, and flowcharts to assist with provision of care and remote patient monitoring during COVID-19 pandemic [4].

Our findings around how clinicians were clustering care or taking short-cuts as a necessity to manage the daily pressures and workload demands adds to the growing body of literature around how nurses clustered care by grouping care activities together [7,9–11,19]. In one study, nurses reported sometimes feeling it necessary to take shortcuts in patient care [24] and a sample of emergency physicians reported taking shortcuts during stressful shifts [25]. Our findings are also similar to what was found in a qualitative study where adaptations and innovations of new processes and resources were made to enable disciplinary and interdisciplinary work and streamline oncology patients’ journeys [26] during the COVID crisis.

Our study highlights how in many situations, the prioritization and adaptation of care resulted in missed or rationed care (washing and hygiene, relational care, turning, mobilizing, and therapy) or delays in care. This finding is echoed in other empirical work including two recent systematic reviews that reported missed and rationed oral care, ambulation, and relational care [27–29]. A scoping review reported the most frequent missed care during the COVID-19 pandemic included oral hygiene, ambulation/mobilization, patient teaching, wound care, and answering the call bell [30]. A comparative cross-sectional study reported that missed nursing care (mouth care, turning patient every two hours, patient teaching, and providing emotional support) increased during COVID-19 [22.8% before COVID-19 to 32.6%. (*P *< 0.001)] [31]. A qualitative study conducted in the ICU revealed that staff shortages during COVID-19 resulted in reprioritization of care that contributed to adverse events [20]. Our findings on the moral distress reported by healthcare professionals as a result of missed or rationed care and the lack of patient and family-centric care is similar to findings from other studies [20,29,32,33]. For example, not providing care to their usual standard had a personal impact on ICU nurses causing them moral distress [20]. Further, one of the systematic reviews reported that rationing or inability to provide care because of resource limitations resulted in nurses experiencing moral distress impacting their home and work-life balance [29].

### Implications

Our study findings underscore the need for leaders to employ more integrated and systematic approaches to managing changes to care and responding to system pressures (e.g., system capacity, patient complexity and volumes). Specifically, leaders need to create healthcare ecosystems that leverage advanced planning [20]; anticipatory strategies alongside on-the-day adaptations [1] including innovative practices; and enhance workforce resilience [4] amid highly pressured situations [4]. Leaders at the clinical microsystem level need to employ a portfolio of strategies when pressures are high (e.g., amid COVID-19 pandemic and our current HHR crisis) by selecting and deploying from the full range of adaptive strategies available [1,4,34,35]. This requires a coordinated strategy to manage the wider contextual pressures (e.g., system capacity, patient volume, and complexity) by adapting ways of working compared to a fragmented and individualised set of improvisations to ensure maximum protection for patients and support for staff [1].

Leaders can draw from the taxonomy of strategies for adapting to pressures developed by Page et al. (2023) to develop actions to guide healthcare professionals at the clinical microsystem level that can be done in anticipation and actions that can be done in daily practice.^1^ To put adaptive strategies into practice and support innovations, there needs to be organizational capacity or ‘slack’^1^ including access to additional resources [1]. This is echoed in the COVID-19 System Shock Framework developed from ethnographic methods that mapped out innovations and initiatives implemented across a pediatric network [4]. Adaptive and flexible organizational structures, effective communication systems, and committed leadership are called for to enable healthcare professionals to create and implement innovations in response to current and future health shocks [4]. Leadership and management support and communication were also identified as a key strategy in the study exploring adaptive models of care in the ICU setting [20].

### Strengths and weaknesses

The key strength of our study is that it involved a large sample and included recruiting from a variety of participants including a range of clinicians/health care providers and those involved in implementing the organizational and unit level practices and policies to guide clinicians/health care providers changes to providing care amid the COVID-19 pandemic and in post recovery efforts. This was a limitation identified in a previous study [17]. Study findings need to be interpreted with the following limitations: the self-reported and subjective nature of interviews and the majority of participating sites being large academic health sciences centre situated within the same large urban city, may limit the transferability of study findings to other contexts.

## Conclusion

Findings from this study elucidated how healthcare professionals adapted and prioritized care at the clinical microsystem level amid several competing pressures. Innovating, clustering care, and taking shortcuts emerged as adaptations undertaken by healthcare professionals to manage their workload, tasks, and demands. In turn, adapting and prioritizing care resulted in missed or delayed care that in some cases evoked moral distress in healthcare professionals, and in some cases, caused harm to patients. Our study calls for leaders to develop and deploy anticipatory adaptive strategies at the organizational level to mitigate pressures related to system capacity, patient volume, and patient complexity. In turn, anticipatory adaptive strategies can guide efforts by healthcare professionals to manage and adapt their clinical tasks, workload, and demands, ensuring patient safety and workforce resilience at the clinical microsystem level. Further research around adaptive strategies, both anticipatory and on-the-day adaptations at the meso and micro levels is needed to inform future post-pandemic efforts.

## Supporting information

S1 File Research Ethics Board (REB) approvals. (PDF)

S2 File Interview guide. (DOCX)

S3 File Narrative dataset. (DOCX)
