# Supplementary material for: Elucidating insights on how care was prioritized, adapted, and missed during and post pandemic
Source: PLoS One. 2025 Jul 17;20(7):e0327464. doi: 10.1371/journal.pone.0327464 (PMC12270122; doi:10.1371/journal.pone.0327464)
Supplement: S1 File — (PDF) [file pone.0327464.s001.pdf]

Human Participants Research Checklist

Complete the following if your study involved human participants or human participants' data. These questions should be addressed for prospective and retrospective studies.

1. Did you obtain ethics approval for this study?

   - If yes, please upload (file type "Other") the original approval document you received from your ethics committee. <u>If the original document is in another language, please also provide an English translation.</u>

     Uploaded

   - If you did not obtain ethical approval, please explain why this was not required below.

2. If you prospectively recruited human participants for the study – for example, you conducted a clinical trial, distributed questionnaires, or obtained tissues, data or samples for the purposes of this study, please report in the Methods:

   i. the day, month and year of the **start and end** of the recruitment period for this study.

   November 29, 2022 – September 7, 2023

   ii. whether participants provided informed consent, and if so, what type was obtained (for instance, written or verbal, and if verbal, how it was documented and witnessed). If your study included minors, state whether you obtained consent from parents or guardians. If the need for consent was waived by the ethics committee, please include this information.

   The participants provided written and verbal consent.

3. If you are reporting a retrospective study of medical records or archived samples, please report in the Methods section:

   i. the day, month and year when the data were accessed for research purposes

   ii. whether authors had access to information that could identify individual participants during or after data collection

   N/A

Sinai Health | Mount Sinai Hospital
Joseph & Wolf Lebovic Health Complex

Research Ethics Board

700 University Avenue, 8th fl., Suite 8-600
Toronto, Ontario, Canada, M5G 1Z5
t: (416) 586-4875 f: (416) 586-4715
www.mtsinai.on.ca

Notification of REB Initial Approval (Delegated)

Date: November 9, 2022

To: Dr. Lianne Jeffs
Department of Research
Mount Sinai Hospital
600 University Avenue Room 20.407
Toronto, Ontario M5G 1X5 Canada

Re: **22-0153-E**
Exploring Evolving Models of Care During the COVID-19 Pandemic (eSubmission)

Sponsor: Canadian Institutes of Health Research (CIHR)
REB Review Type: Delegated
REB Initial Approval Date: 09 November, 2022
REB Expiry Date: 09 November, 2023
Documents Approved: Protocol (Dated: 2022/11/09)
Consent Form (Dated: 2022/10/03)
Appendix A: Email Recruitment Letters (Date: 2022/06/30)
Appendix B: Participant Demographic Data Collection (Dated: 2022/06/28)
Appendix B(2) Data Collection Spreadsheet (Dated: 2022/06/28)
Appendix C: MoC Interview Guide (Leadership/Decision Maker) (Dated: 2022/06/30)
Appendix D: MoC Interview Guide (Implementation Team) (Dated: 2022/06/30)
Appendix E: MoC Interview Guide (Point of Care Team) (Dated: 2022/06/30)
Appendix G: Verbal Consent Checklist (Dated: 2022/06/28)
Appendix H: Recruitment Poster (Dated: 2022/10/03)
Documents Acknowledged: N/A
Health Records Access: No

The above named study has been reviewed and approved by the Mount Sinai Hospital Research Ethics Board. If, during the course of the research, there are any serious adverse events, confidentiality concerns, changes in the approved project, or any new information that must be considered with respect to the project, these should be brought to the immediate attention of the REB. In the event of a privacy breach, you are responsible for reporting the breach to the MSH REB and the MSH Corporate Privacy Office (in accordance with Ontario health privacy legislation – Personal Health Information Protection Act, 2004). Additionally, the MSH REB requires reports of inappropriate/unauthorized use of the information.

If the study is expected to continue beyond the expiry date, you are responsible for ensuring the study receives re-approval. The REB must be notified of the completion or termination of this study and a final report provided. As the Principal Investigator, you are responsible for the ethical conduct of this study.

The MSH Research Ethics Board operates in compliance with the Tri-Council Policy Statement 2, ICH/GCP Guidelines, Part C, Division 5 of the Food and Drug Regulations of Health Canada, Part 4 of the Natural Health Product Regulations, and Part 3 of the Medical Devices Regulations.

During the COVID-19 Publicly Declared Emergency, the REB continues to review and approve submissions, but initiation or implementation of newly approved submissions will be contingent upon evolving institutional policies and guidelines. Principal Investigators are encouraged to consult with their Department Heads for further guidance.

Sincerely,

Vibhuti Shah, MD, FRCPC, MSc
Chair, Mount Sinai Hospital Research Ethics Board

Sunnybrook
HEALTH SCIENCES CENTRE

To: Ms Tracey Das Gupta
From: Dr. Brian Murray
Date: 22/Nov/2022
Subject: Exploring Evolving Models of Care During the COVID-19 Pandemic

Project Identification Number: 5571

Approval Date: 22/Nov/2022

Expiry Date: **22/Nov/2023**

The Research Ethics Board of Sunnybrook Health Sciences Centre has conducted a delegated review of the research study referenced above and approved the involvement of human participants. REB members involved in the research study or who have declared a conflict do not participate in the review, discussion or decision. All other required institutional approvals must also be obtained prior to the conduct of the study at this research site.

Approved Documents:

Document Name	Document Date	Document Version
Protocol	19/Sep/2022	
Verbal Consent Checklist	19/Sep/2022	
Data Collection Sheet	23/Aug/2022	
Interview Guide	30/Jun/2022	
Recruitment Email	23/Aug/2022	
Recruitment Flyer	23/Aug/2022	
Consent Form	30/Oct/2022	2

Ensure that study advertisements and/or posters are submitted to Communication and Stakeholder Relations for review prior to posting.

As Principal Investigator you are responsible for the ethical conduct of this study which may be subject to review by Quality Assurance and Education. The study must comply with current legislation outlined in the Ontario Personal Health Information Protection Act (PHIPA) and all acts, regulations, guidelines and policies that govern this research. Note that regional or provincial 'shared systems' that may be accessible from Sunnybrook systems (e.g. cGTA/Connecting Ontario from SunnyCare) cannot be used for research purposes (unless approved by the institution in limited circumstances).

The REB requires immediate notification of internal serious adverse events and significant deviations, submission of a renewal form prior to the approval expiry date, and notification of study closure.

All required institutional approvals must be obtained prior to the conduct of this study at Sunnybrook (e.g. including but not limited to a completed Study Impact Approval form, regulatory approvals from Health Canada, contracts etc. as applicable).

The REB and Research Ethics Office are in support of facilitating the progress of ethical research and thank you in advance for your efforts to study research participants. Best wishes for a successful study.

Brian J. Murray, MD FRCPC FAAN D, ABSM
Chair, Research Ethics Board

The Research Ethics Board of Sunnybrook Health Sciences Centre Operates in Compliance with the Tri-Council Policy Statement 2nd

edition, ICH GCP Guidelines, Part C Division 5 of the Food and Drug Regulations, Part C Division 3 of the Food and Drug Regulations, Part 4 of the Natural Health Products Regulations, Part 3 of the Medical Devices Regulations, and the Provisions of PHIPA 2004 and its applicable regulations. The Sunnybrook Research Ethics Board is registered with the U.S. Department of Health and Human Services (DHHS) Office for Human Research Protection (OHRP). All Health Canada regulated trials at Sunnybrook are conducted by a Qualified Investigator.

Fully affiliated with the University of Toronto

University Health Network
Research Ethics Board
700 University Ave, 4th Floor
Toronto, Ontario, M5G 1Z5
Phone: (416) 581-7849

NOTIFICATION OF REB INITIAL APPROVAL

Date: July 11, 2023

To: Pam Hubley
Toronto General Hospital, 200 Elizabeth St., Toronto,
Ontario, Canada, M5G 2C4

Re: 23-5214
Exploring Evolving Models of Care During the
COVID-19 Pandemic

REB Review Type: Delegated
REB Initial Approval Date: July 11, 2023
REB Expiry Date: July 11, 2024

Documents Approved:

Document Name	Version Date	Version ID
UHN Recruitment Flyer	June 6, 2023	1
Recruitment Emails	June 20, 2023	2
Protocol	March 9, 2023	4

Documents Acknowledged:

Document Name	Version Date	Version ID
Consent Form (Mount Sinai)	July 5, 2023	3
Amendment MSH REB approval	July 6, 2023	

The University Health Network Research Ethics Board approves the above mentioned study as it has been found to comply with relevant research ethics guidelines, as well as the Ontario Personal Health Information Protection Act (PHIPA), 2004.

The study is approved as applicable to UHN site participation.

Best wishes on the successful completion of your project.

Sincerely,
Morris Sherman
Co-Chair, University Health Network Research Ethics Board
Approved and Digitally signed by Morris Sherman on July 11, 2023 at 08:17 PM

The UHN Research Ethics Board operates in compliance with the Tri-Council Policy Statement; ICH Guideline for Good Clinical Practice E6; Ontario Personal Health Information Protection Act (2004); Part C Division 5 of the Food and Drug Regulations; Part 4 of the Natural Health Products Regulations and the Medical Devices Regulations of Health Canada. The approval and the views of the REB have been documented in writing.
Furthermore, members of the Research Ethics Board who are named as Investigators in research studies do not participate in discussions related to, nor vote on such studies when they are presented to the REB.

UBC-Providence Health Care Research Institute
Office of Research Ethics
10th Floor Hornby Site - SPH
c/o 1081 Burrard St.
Vancouver, BC V6Z 1Y6
Tel: (604) 806-8567
Fax: (604) 806-8568

Providence Health Care Institutional Certificate of Final Approval

PRINCIPAL INVESTIGATOR:	DEPARTMENT:	UBC REB NUMBER:
Agnes Black	UBC/Applied Science/Nursing	H22-02792

SPONSORING AGENCIES:
N/A

PROJECT TITLE:
Exploring Evolving Models of Care During the COVID-19 Pandemic

Ethics Certificate Released:
January 6, 2023

PHC Institutional Approval Date: January 6, 2023

The UBC-PHC Research Ethics Board granted ethical approval for the above-referenced research project on the date stated above. All necessary hospital department/facilities approvals and institutional agreements/contracts are now in place and you have permission to begin your research. *

Dr. Darryl Knight

VP Research & Academic Affairs, Providence Health Care
President, PHC Research Institute

* PHC Health Information Management requires a copy of this certificate prior to granting access to records.

St. Paul's Hospital
Holy Family Hospital
Mount St. Joseph's Hospital
St. Vincent's Hospital-Brock Fahrni Pavilion
St. Vincent's Hospital-Langara
Youville Residence

SHN SCARBOROUGH HEALTH NETWORK
RESEARCH INSTITUTE

Scarborough Health Network
Research Ethics Board

To: Minette MacNeil

SHN REB File No: MIS-23-006

Study Title: Exploring Evolving Models of Care During the COVID-19 Pandemic

Re: Study Approval

Approval Date: June 7, 2023 to June 7, 2024

Review Type: Delegated Review

Documents Acknowledged:
- MIS-23-006 MoC_REB Application Form_v1.0_17Apr2023
- MIS-23-006 MoC_Protocol_v1.1_30Jun2022
- MIS-23-006 MoC_Appendices C, D, E - Interview Guide_v1.3_30Jun2022
- MIS-23-006 MoC_Appendix A - Recruitment Email_v1.0_15Aug2022
- MIS-23-006 MoC_Appendix B - Data Collection Sheet_v1.0_23Sep2022
- MIS-23-006 MoC_Appendix G - Verbal Consent Checklist_v1.0_23Sep2022
- MIS-23-006 MoC_Appendix H - Recruitment Flyer_v1.0_23Sep2022
- MIS-23-006 MoC_Appendix H - Written Consent Form_v1.0_19Sep2022
- MIS-23-006 MoC_JeffsL_Academic CV_01Jan2023
- MIS-23-006 MoC_SHN REB Response and Revisions Letter_16May2023
- MIS-23-006 MoC_Protocol_tracked_v1.2_12May2023
- MIS-23-006 MoC_Protocol_clean_v1.2_12May2023
- MIS-23-006 MoC_Appendix A - Recruitment Email_tracked_v1.1_12May2023
- MIS-23-006 MoC_Appendix A - Recruitment Email_clean_v1.1_12May2023
- MIS-23-006 MoC_Appendix B - Data Collection Sheet_tracked_v1.1_12May2023
- MIS-23-006 MoC_Appendix B - Data Collection Sheet_clean_v1.1_12May2023
- MIS-23-006 MoC_Appendix F - Written Consent Form_tracked_v2.0_12May2023
- MIS-23-006 MoC_Appendix F - Written Consent Form_clean_v2.0_12May2023

Scarborough Health Network (SHN) Research Ethics Board (REB) has received, reviewed the above documents and granted <u>**Approval**</u> to the above titled study.

Please note that it is your responsibility to ensure in future that request for re-approvals be submitted prior to the date.

Should this study continue beyond the expiry date noted above, you must submit a request for re-approval prior to this date. Please advise the REB annually on the progress of your research. During the course of the research, any significant deviations from the protocol or any adverse events should be brought to the attention of the REB. If the study has been completed by this date, a Completion Form should be submitted.

The SHN Research Ethics Board operates in compliance with the Tri-Council Policy Statement; ICH Guideline for Good Clinical Practice E6(R1); Ontario Personal Health Information Protection Act (2004); Part C Division 5 of the Food and Drug Regulations; Part 4 of the Natural Health Products Regulations and the Medical Devices Regulations of Health Canada. The approval and the views of the REB have been documented in writing. Furthermore, members of the Research Ethics Board who are named as Investigators in research studies do not participate in discussions related to, nor vote on such studies when they are presented to the REB.

SHN

Nisanthini Ravichandiran
Nisanthini Ravichandiran (Jun 9, 2023 22:44 EDT)

Dr. Nisanthini Ravichandiran, MD, CCFP
Chair, Research Ethics Board
Scarborough Health Network

Jun 9, 2023
Date

Research Ethics Office
Telephone: (416) 864-6060 Ext. 42557
Facsimile: (416) 864-6043
E-mail: Dharmista.Patel@unityhealth.to

UNITY HEALTH TORONTO

July 25, 2023

Dr. Alexandra Harris,
St. Michael's Hospital

Dear Dr. Harris,

Re: REB# 23-058[C] - Exploring Evolving Models of Care During the COVID-19 Pandemic

**REB APPROVAL:**	**Original Approval Date**	**July 25, 2023**
	**Annual/Interval Review Date**	**July 25, 2024**

Thank you for your application submitted on **March 9, 2023**. The above noted study has been reviewed through a delegated process (not by Full Board review). The views of the Unity Health Toronto Research Ethics Board (REB) have been documented and resolved. Please note that no member of the REB associated with this study was involved in its review or approval.

The REB approves the study as it is found to comply with relevant research ethics guidelines, as well as the Ontario Personal Health Information Protection Act (PHIPA), 2004. The REB hereby issues approval for the above named study for a period of 12 months from the date of this letter. Continuation beyond that date will require further review of REB approval.

In addition, the following documents have been reviewed and are hereby approved:
1. Protocol - ver: 02-May-2023
2. Appendix A: Recruitment Emails ver: 15-Feb-2023
3. Appendix H: Recruitment Flyer ver: 15-Feb-2023

Furthermore, the following documents have been received and are acknowledged:
1. Appendix B: Data Collection Sheet ver: 15-Feb-2023
2. Appendix C, D, E - Interview Guide ver: 01-May-2023
3. Appendic F: Consent Form - Mt. Sinai ver: 3 09-Mar-2023
4. Other REB Approval Letters - Athabasca University ver: 01-Dec-2022
5. Other REB Approval Letters - Providence Health Care ver: 06-Jan-2023
6. Other REB Approval Letters - Mount Sinai Hospital ver: 09-Nov-2022
7. Other REB Approval Letters - Sunnybrook ver: 22-Nov-2022

This approval is for the referral of potential participants to the lead Principal Investigator Dr. Lianne Jeffs (Mt. Sinai Hospital). As stated in the submission, all study-related conduct, including the consent process will take place at Mt. Sinai Hospital.

Please note that all clinical research studies are required to comply with the institution's guidelines related to on-site and in-person research. This REB approval letter does not override any restrictions covered in those guidelines.

During the course of this investigation, any significant deviations from the approved protocol and/or unanticipated developments or significant adverse events should immediately be brought to the attention of the REB. Please note that shared electronic health systems such as ConnectingOntario, PRO, RM&R, OLIS, HDIRS, eCHN, DPV and IAR do not permit access for research purposes.

For all interventional studies, the study sponsor is required to register the study in a publicly accessible registry prior to enrollment of the first study participant. The study sponsor is also required to ensure that the trial record is updated in a timely manner and the summary results are posted within the required timelines.

All institutional approvals **must be** coordinated and approved through the Office of Research Administration (ORA) prior to initiation of this research. If a Clinical Trial Agreement is required, it must be reviewed and approved by the ORA, prior to commencing any study related activities.

All investigational drug product dispensing must be coordinated through the Research Pharmacy.

REB operates in compliance with the Tri-Council Policy Statement Ethical Conduct for Research Involving Humans, the Ontario Personal Health Information Protection Act, 2004, and ICH Good Clinical Practice Consolidated Guideline E6, Health Canada Part C Division 5 of the Food and Drug Regulations, Part 4 of the Natural Health Product Regulations, and the Medical Devices regulations. Furthermore, all investigational drug trials at Unity Health Toronto are conducted by Qualified Investigators (as defined in the latter document).

With best wishes

☐ **David Mazer, MD, Chair, REB** ☐ **Michael Szego, PhD, Vice Chair, REB**
☐ **Melanie Tsang, MD, Vice Chair, REB** ☐ **Zoe von Aesch, MD, Vice Chair, REB**
DM/MS/MT/ZVA/aml
