# Supplementary material for: Elucidating insights on how care was prioritized, adapted, and missed during and post pandemic
Source: PLoS One. 2025 Jul 17;20(7):e0327464. doi: 10.1371/journal.pone.0327464 (PMC12270122; doi:10.1371/journal.pone.0327464)
Supplement: S2 File — (DOCX) [file pone.0327464.s002.docx]

**Supplementary File 2 – Interview Guide**

**Change to care (est. 8-10 min)**

1. What was your role like before the pandemic?
2. How did care change over the course of the last 3 years? What did you do differently?
3. What is providing care like now?

**Provisions of care (est. 8-10 min)**

1. What did you/your team stop doing?
2. Can you describe an experience where you were not able to provide the care that you wanted to?
3. How did you/your team prioritize care?

**Context of care/How did changes feel? (est. 5-7 min)**

1. Reflecting on the past three years, how did the care change feel for you/your team?
2. What supports were available to you? From leaders? From managers? Organizationally?

**Implementation/Evaluation (est. 10 min)**

1. How were the changes implemented?
2. Were you aware/involved in the implementation? If so, how?
3. How was it communicated to you/your team? How did you learn/teach information about the changes/provisions? How were these practises socialized to you/your team?
4. How were changes to care evaluated/reviewed?
5. What barriers need to be dropped for you to provide quality care? OR what can facilitate quality care for you?

**Innovation/Forward/Future Thinking (est. 10-15 min)**

1. Can you share what innovations in care/improvisations in care that you were part of, or heard about over the past three years?
2. Reflecting back on the past three years, what recommendations do you have to move forward, to ensure that quality care is provided?
