# Supplementary material for: Elucidating insights on how care was prioritized, adapted, and missed during and post pandemic
Source: PLoS One. 2025 Jul 17;20(7):e0327464. doi: 10.1371/journal.pone.0327464 (PMC12270122; doi:10.1371/journal.pone.0327464)
Supplement: S3 File — (DOCX) [file pone.0327464.s003.docx]

**Supplementary File 3 Changes to Care Codebook**

| **Theme** | **Supporting Narrative** |
| --- | --- |
| Prioritizing care based  on system capacity, patient volume and complexity | Trying to think about ways that we could ensure that care didn't get disrupted even if we're going to shift to a different model. As well as in my inpatient units, we basically decided that we needed to stop the inpatient program and then become an alternative level of care. So that we could start to decant other areas of the hospital to support the other areas that our program was basically closed. [Site 1 001]  The models of care, everything was slowed down because every room was basically isolation … The OR was pretty much shut down. Any surgeries that weren't emergent during that wave to because we took over a lot of the cardiac surgical ICU, all of the cardiac ICU, two HAUs, and PAR I guess was technically our next step, but we never really got there. PAR being just recovery room. HAU being high acuity unit. So, yes, it definitely slowed the OR down. [Site 1 003]  A lot of our heart centre program was also a lot of outpatient activity. Which was put to a halt, essentially….Just prioritising certain things - all the bigger projects were on hold, the new x’s project, and some other strategic direction projects. [Site 1 005]  I went from 100 percent in person work type to overnight shutting down our clinic. We shut down basically over night. So we had to pivot over night over the weekend to virtual care. So apparently our organization was able to organize virtual access to all the things that we needed, we were just not permitted prior to the pandemic to work from home. [Site 1 013]  It was the people who really needed us and were motivated to see us that we would prioritise. [Site 1 016]  If it was not an emergency, it didn’t come through the door. But there were some provisions made to doing urgent patients. So, for example, cancer patients. We got through most cancer patients within a reasonable time. Everything else was just an emergency during the COVID. [Site 1 028]  We got some input from the pharmacist, and we developed here’s the top priority things you’ve got to do, no one is going to argue with you that you got to do these things. Then if you have time, do these things. Low level priority things - don’t worry about these things. We helped developed an algorithm when you’re really busy, here is what you should focus on and truly don’t worry about these things that you would otherwise would’ve done for sure. The other thing we did was we came up with contingency planning, the leadership team really worked hard to say okay, if we’re down this many pharmacists, what do we do? [Site 2 002]  Prioritize the skin, skincare, mouthcare. But we still tried to make sure the patient looked nice as a human. But if you want to do it to yourself, you will do some shortcuts. [Site 2 006]  We couldn't do home assessments, for the longest time, because of the pandemic. So we were counting a lot of pictures from family members and things like that. [Site 2 022]  Right at that moment they also decided to slowly stop doing elective surgeries [Site 2 023]  We actually sat down to look at what we could eliminate, charting documentation, can we not do a Braden today, can we – a falls risk assessment. Can you not do – oh, you know, does it need to be done every day or can we do it every other day? Do we need to have bed baths? Things like that. And that was important just to relieve the stressors on some of my new staff but also some of the burnout for staff who were, you know, probably over census or over capacity. What did it look like on our unit? We basically looked at all the documentation. Said, you know, if you don’t need to do certain things, certain – we went through, the CNS and I, we went through all of the documentation our nurses do every day. Very much made a list of things that were first priority, second priority, third priority. It was a day-to-day discussion. So we didn’t know what shift would look like, the next shift would look like. If we get a sick call do we, you know, can we replace with overtime? Can we replace with a floating a nurse from another unit? … I think the provisions of care was more a reaction or a strategy based off of the pressure. I would say the pressures of the system were always present. They got worse over time. Provisions of care and lots of care came into being as a way to mitigate risk and continue to provide care in response to those stressors. [Site 2 024]  Because you now we changed our provision of care. So that was a little bit hard to adjust to because we’re used to providing a certain standard of care, and so kind of going below that standard was a hard adjustment. I am having a hard time going back to my previous standard of care. So I feel like I am operating at the low of what I used to do just because of the increased workload and the higher turnover. [Site 2 031]  I think the whole notion of provisions of care is important too, is that you know, we’re inevitably going to come into situations where we’re seeing surges in patients, or staffing challenges. And we may have to consider, you know – and from a risk perspective, if there’s some sort of critical event, it’s a way of documenting that, you know what, we actually were in a really, you know, crisis situation – well I think it was contingency crisis, right? so we were in a contingency state, where we knew that we were having to manage more than we could, based on our – sort of our unusual staffing models, or resources that – available. And because of either the increased volume coming in, or the decrease resources, right? So I think that that’s actually – having that as a tool, is also value add for going forward, for sure. [Site 2 033]  So we had to cancel – so we have our infectious disease clinic where the doctors see patients Monday to Wednesday and sometimes on Thursday, typically until noon, sometimes into 2:00. But we had to cancel all of the infectious disease doctors' clinics during that time. So they weren't onsite, they weren't here, they weren't seeing patients. And so then for all of the patients who were cancelled [Site 3 003]  I just kind of prioritise what was going to be the most essential things to bring in. So I just bring in a guitar, maybe a drum and an iPad with speakers, so that again, because trying to keep a little distance, volume became an important piece. Yeah, that was basically it, just prioritise the items that I felt were essential. And also prioritise going and speaking with people. Our team Creative Arts Therapies, we're all registered psychotherapists. So, especially during this time, I think we all felt it was really important that we provided a counselling aspect as well. And especially since people couldn't really see their families just to provide company as well. So obviously, sometimes when there’s an outbreak or anything, I’d just bring nothing in and just go sit and talk. [Site 3 006]  It was challenging in a sense of that you had to prioritize your workload very carefully. I would say that it was difficult to sometimes go above and beyond for these patients when they when – they needed it, and you want to provide that type of care. I think sometimes spending more time with these patients was hard to do because of the workload and the acuity. I would say prioritizing was a big part of how to go about the care. So the rapid nurse and I would kind of flag certain patients and then I would have a list of the patients and almost like a spreadsheet of what needs to be done for that patient, the plan, the settings, and I guess any additional comments. So I would prioritize by seeing the most sickest patients and the more – like at first and then and then go from there. So any issues that I had, I would either touch base with rapid, critical care, or the MRP to flag any issues at the time. I think I was able to provide that type of care sometimes at the cost of my own well-being. I provided that and I did whatever I could for the patients at the time. Yes, and I think I can – I can say a lot of health care workers probably did the same where they provided the care that was needed and deserving of that patient and at the point of sometimes sacrificing their well-being – their own well-being for that patient [Site 3 009]  The most symptomatic patients were prioritized and seen obviously first and managed more acutely. Those that were, you know, more stable may have not gotten a check-in on a daily basis; would have gone a check-in, you know, every second day. We would rely on the primary teams to see if things are unstable call us back. So prioritizing the more acute patients. [Site 3 012]  What are my most priority? What are the things that have to be done that I cannot not do which are my safety? And all of those checks that just need to be done, like, there's no second guessing that. After that, sort of the other elements of care, I would try to get, like utilize, if I had the model with a buddy nurse, I would utilize them for some of the, like, some of those aspects of care. So, like, maybe you can like, help brush teeth or something like that or help, you know, change this dressing or something small, like the smaller things. I think just because of it being COVID, we tried to bundle our care as much as we could, that was just something that was sort of easier for us to do. So, prioritizing really what they needed, sort of bundling the care. Otherwise, I mean, really just being available to help where you could and knowing where the needs were in the unit, that was the most important thing. But the thing is, is I think depending on what type of nurse you are, like, if you're the type that just wants to sit there and be with your assignment, that's one thing. [Site 3 017] |
| Adapting care by innovating, clustering, and taking shortcuts; | - There was lots of changes related to the PPE, the design of the units, making sure that we had PPE making sure that we had a process for monitoring and getting supplies for PPE. The patients had their IV poles outside of the private rooms if they were COVID-19, so that nurses could easily up titrate and down titrate pressors and isotopes, which was way different than previously. COVID allowed us to be able to do things and test things out prior to the new x. So, for the new there was obviously a valid reason for us to come together and to collaborate to share our resources and make decisions with a broader lens across all of the areas as opposed to each area making decisions based on what's going on within their unit. It allowed us to do a lot of testing actually, and early implementation strategies that we would have wanted to do leading up to the new x and the critical care complex. So, I think we're well ahead of the game in preparation, because of the COVID pandemic. [Site 1 002] - Many time we were doing shortcuts just to make sure like things were done and sometimes these were against hospital policies, but if you didn’t do these shortcuts you wouldn’t like do everything that you needed in that shift, that were necessary for patient care. For example, let’s say you were giving a patient medications – they can’t swallow the medications, they have a tube, through their nose or gastric tube, policy would be you would crush each medication separately and administer them one by one, but that could take a real long time if they’re taking 16 medications in the morning, so we’d like crush all of them and put them in one cup and then give it all at once. [Site 1 004]   Tried to be open minded. It’s a different situation and I have to learn to be adaptive. This is what I learned through all this year. I try to be open and what is needed then do it. And it can – it doesn’t have to be the same but we need to try. It cannot be just a box that “Oh we did that before, we cannot do it now.” We have to be flexible for patient or for team members, we just have to be – we will go through this every one of us has to just step out of our comfort zone. [Site 1 010]   - It was a chaotic time where we had to kind of just do it on our own and trial and error. And then as x came in then we adapted what we were doing based on their guidelines or adapted our documents based on their documents they had provided So what can we do to ensure that these patients are seen because we know that elective surgeries aren’t going to be done any time soon right now. So how can we split this and make sure that we’re seeing patients now versus them sitting out there on their own at home deteriorating functionally, right. [Site 1 017] - As far as nurses being innovative with ways of making sure we didn't have to go in the rooms as much, it was definitely piggybacking infusion meds. It was safe. It wasn't a bad thing or anything. I was saying about anterooms having the extension tubing so that you didn't have to go into the rooms to deal with the pumps. - We were clustering our care.. ... [Pause] No, but just to give you an example, if we had, say someone was on an IV antibiotic every four hours, that's not something that you cannot go in four hours. That's not something that you cannot go in for. So we would try, and bring all our supplies, and get all our meds. And we'd go in in the morning and then do our assessment and do all the turns. We would try and get it all done in that first hour or so because you know once you go out, you try and get blood work done maybe a little bit early, or maybe a little bit late because you are trying to push it for when you're going back in the room. Does that make sense? [Site 1 027] - We were all making these wild adaptations [PHC …] - Many people experience in pharmacy and outside of pharmacy, but we didn’t have a set of instructions to follow and we’ve never done it before. We were making it up in the moment, which is never a great system, of course. I don’t think we even really had enough time to figure out if we were doing it the right way or not. Yeah, I’m probably shouldn’t sugar coat it, we were in panic mode so much of the time and ideally we would’ve had weeks to sit down and talk with everybody and say we could do it this way or we could do it that way or should we form a committee. But it was really on the fly, we started an email distribution chain, we made some plots and tried to figure out ways people could use the information and over time more and more people wanted to be part of the email chain to get the information. [Site 2 001] - During COVID, we tried to wash them thoroughly but during COVID because you have extra things to do and extra responsibility, you only have to wash whatever is important. [SH006] We were deployed to a nursing home and the PSW or the nurse, [so as we basin] that oh, we don’t need a basin. We’re so short so just do the face and the private care. I’m not going[to wash their armpits. So we did some shortcuts. When I do my care, I always make sure at least my patient look nice, the face, they don’t smell. But shortcut, it’s just like you’re used to washing them using four towels, or face cloths, you just have to do it with three face cloths. Just do the best you can to get them ready for the day. Today I washed the patient thoroughly, and the second day I don’t have to spend that much because I have to do some procedures that is more important than washing the patient. The only shortcuts that I will do is washing the patients. Instead of washing the patient all over, I have to skip washing the chest, the back, the legs, something like that. Because hair care is very important. [Site 2 006] - Within that team we were problem solving things together, it's very real time. Situations would arise and we had to think about, “OK how do we address this so it doesn’t happen next time?” So you were problem solving on the go. [Site 2 016] - We were notified hospital-wide programs are suspended and that you can implement one-to-one therapy, and we were lucky in that we were also permitted to run small, like, up to five patients within a group, but it had to be unit-based only. And there were a lot of also precautions within the group. Patients had to be six feet apart. So, programs like games night, for example, playing dominoes really wasn’t – we had to really think about programs where patients could actually be six feet apart. So, that was another precaution. And also, the one thing that changed is no singing. So, as you can imagine, recreation and leisure really utilizes music as an intervention. [Site 2 030]   We had a meeting and kind of streamlined and I guess changed the provisions of care of like what our bare minimum would be when we are at like low staff or when the surge is too high or something like that. Yes, so before COVID I would always talk to the patient and the family and then during COVID I resulted in just talking to one or the other most times, just because there wasn’t enough time to have all those conversations. Also I guess like checking in on the patients during their stay it reduced a little bit during COVID and the search times. Like I mentioned, it kind of resorted to most of my work being when they were admitted and when they’re being discharged. And then if I was able to or if I had capacity then I would check in during their stay to see if they had any questions. Or course for patients who it’s more complicated and we’re making changes to their regimen and I need to do the follow-up, I would prioritize those and continue to check in on those patients. For the ones who are more stable, I wouldn’t be checking in on them as often, just by way of prioritizing and triaging their needs. [Site 2 031]   - We really had to think outside the box and really problem solving. I feel that it's more case to case tailor made care plans for patients during the pandemic, versus you kind of – generally most patients, we can just send a referral, and then they'll follow up…. So just in partnership with our volunteers, we have an activity box on the unit for colouring or word searches or other like mindfulness colouring, and also just stress relieving IPAC safe toys that can be viroxed, like stress balls, things like that, and soakers. We would just give them an activity package, just to help them pass time. [Site 2] - The strategies I used was really to speak very slowly. When I spoke slower and clearly I found that they were able to understand me better, putting – I also sometimes had to actually take the patient into a different private space just to minimize surrounding environment noise because the hospital itself is very noisy…So the people who were COVID-positive, they would only get therapy at the end of the day. And regardless of how they were during the day, like, normally we prioritize them and try to accommodate based on, say, their energy level or their preferences or what certain medications are given at a certain time to optimize them for therapy. That was all out the window because for the mere fact they were COVID-positive. And so that kind of naturally bundled my day up. For everyone who was COVID-positive they were at the end of the day. And unfortunately if they had to have their therapy at the end of the day and it wasn’t optimal for them, then they didn’t do as well, unfortunately, because we just had to continually see them at the end of the day. I think just the biggest thing is that bundling of care. I think that’s the only part that still carries over now – where it’s trying to be the most efficient with your time with a specific person to be able to disseminate as much information in one go as opposed to returning back to that patient later on, even if other information comes up or such just trying to really keep everything to spurts of interaction with the same person. I would say that’s the only part that’s carried over. [Site 3 001] - It was a Massimo Bluetooth SAT probe that monitor their oxygen level, their heart rate – like their pulse and then their respiratory rate as well. They had like a little Bluetooth device on the patient that was very, very sick and they had the screen and the monitor outside. - … Everyone’s ability to adapt and do what they had to do with the patient at the time I think went very well. Considering how busy it was, how often things were changing, and how intense and overwhelming it could be for the health team, the health care professionals, I think everyone’s ability to adapt and just kind of do what was needed for the patient was amazing. And times we would be proning like six patients in the ICU and people just did what they had to do. It was nice to see that everyone kind of came together to do what they had to do and the type of work that they had to do. Yes, so I think everyone coming together I think and working as a team went pretty well, considering how things were changing very drastically, yes. [Site 3 009]   There was some publication that came out of this, to share it with the broader community that the restrictions created barriers and that barrier was a religious barrier for some people. It did create an opportunity to do some academic work to share this information with a broader audience. It’s keeping the lines of communication open with, you know, the units and documentation and letting, you know, the teams that are involved with the patient understand what the family is dealing with. Well, the shortcut would be that you weren’t doing the emotional support that perhaps would typically happen when you’re fully staffed and you have more time. You were doing very much the physical aspect, the check-in, making sure the symptoms were well managed. But you weren’t necessarily spending time doing more emotional support and being present as much as you would have in times where, you know, you had the staffing. I think really because part of our work as I said earlier is part of being present, bearing witness and providing that emotional component, and that requires time – you can’t rush that part. The psychosocial help that you are able to provide patients and families. I have to say that probably suffered. [Site 3 012]  Patients were going home without the equipment that they needed, and we were having to be creative about then giving them strategies that we would never otherwise give. If you don’t have a wheelchair, here’s a thing, you could try using a walker with a seat and you sit on it, and if you have two family that can help push you up one or two steps, you could try that. I’m not telling you that this is the way to do it, but, you know, to optimize your safety for going home, if this is what you want to do, you could try it this way…Our social worker literally almost ended up with a neck injury from holding it up so much. So, she whipped up this homemade cardboard iPad holder, it worked so well, and then she reinforced it with duct tape and all that. It worked so well that I shared it with my OT colleagues. [Site 3 013]  If I had COVID-positive patients, I would leave those to the end of my rounds, so I wasn’t entering into that room at the beginning, as was recommended. And used kind of the – you know, in terms of my approach to patient care, I would write orders that would support the least amount of contact that nurses need really, going in, providing medications. I would be thoughtful about what I needed to order and how, to kind of limit the interactions based on medications I was ordering. And based on other medications that were already ordered. I tried to coordinate with that if that was at all possible. [Site 3 016]  Because of it being COVID, we tried to bundle our care as much as we could, that was just something that was sort of easier for us to do. So, prioritizing really what they needed, sort of bundling the care. Otherwise, I mean, really just being available to help where you could and knowing where the needs were in the unit, that was the most important thing. But the thing is, is I think depending on what type of nurse you are, like, if you're the type that just wants to sit there and be with your assignment, that's one thing. [Site 3 017]  I’ve had to learn to improvise, put it that way. I use the wipe off board and I’ll write and I’ll hold it up to the camera and say, “I don’t have an appropriate pictogram for what I want to say,” and, you know, make it so it’s a yes/no answer for them. So I think that COVID has revolutionized social work intervention. [SB014]   - We weren't able to get cabbie wipes. So we actually had to create a process of soaking reusable towels. It was creating something that people were like, why are we doing this? Why are nurses doing this because of a supply chain issue? That was just kind of a process admission piece. [Site 3 023] - In terms of what shifted is we really helped to support connecting our patients with their families, and we utilized technology, so we utilized iPads that were provided by the hospital, cellphones were also provided, and TR wasn’t just involved, I would say it was primarily SLP and social work, there were – I’m sure there were other health disciplines involved, but primarily it would be – I just remember collaborating more with CDAs, SLPs, social work on the Zoom calls. The one thing that also stopped, because a lot of the caregivers weren’t coming in, was there’s some patients who need assistance with feeding, and so, we helped with that. And so, that was sort of something outside of our scope of practice, so I would say that was another shift. And so, that was kind of considered part of the alternate model of care for us. So, the one-to-ones, I would say, is appropriate, that’s sort of within our scope. But the feeding would be outside of our scope. [Site 3 030] |
| Being impacted by prioritized and adapted care | Over time some of the more basic nursing practice have fallen to the wayside. Overall, there's less connectivity, there's also been a huge backwards movement regarding patient and family centred care. Now we're at the place where nurses think that it's normal for families to only be allowed at the bedside for a limited amount of time, where that used to never be the case, like families that were entitled to be at the bedside, to visit and there's much more scrutiny around that. Families have less access overall to their loved ones when they're in critical care...That led to delays and patient care, because there were so many people being involved in trying to manage different situations that previously were never involved. That the people who were really positioned to do the work were not allowed to just do the job that they've been doing forever. [Site 1 002]  When you’re working as short as that, medications were given late. Procedures were done late just because you didn’t have enough time to give them…I was in the room with a patient when it was absolutely necessary; doing my assessments, giving medications. Before the pandemic I would have more time and I was like less afraid of catching COVID so I could like actually sit by the patient and give them therapy and communication, listen to their concerns more; just making them feel less alone in their hospitalization. Yeah, just less time at the bedside and I felt like we weren’t meeting like a patient’s social needs or emotional needs, especially like the ones that had no friends or family and they were just very lonely… Some duties that were not [unintelligible] only nurses like we would pass onto like the care aides to do, like changing patients, bathing them, feeding them. [Site 1 004]  I’ve been waiting for my pain medication” or “I’ve been waiting for this.” and I was just “I totally understand but the patient’s very sick, you’ll have to wait.” So that was tricky because it was just like we had to – even how I practice I would have to cluster my care with my COVID patients. I would sort of pop my head in, or if they would call I could chart that, like I’m communicating with them but I would try to save them for last. Our COVID patients sometimes would say, they’re like, “I feel so lonely, like how come you’re not in here with me for –” like, “How come you – how come you guys are very quick in and out?” And I was just like, “Well we can’t be here with you. We just –” and you didn’t – you had to be cautious with how you said that because – how you would communicate that because you don’t want them to feel neglected. Things do fall behind, you can’t do a lot of the extra tasks that can unfortunately wait such as maybe hygiene, or making their beds, or maybe you are late a little bit with their medications. And it’s hard to explain that to patients because if you are being honest, and you’re like, “Well there’s a patient who’s sicker and we just have to all support each other.” Most of the time they find that insulting. from a patient’s perspective, if they’re, you know, cognitive, and they’re lucid, and they just need some help with washing their face or, you know, hygiene, or they want to go for a walk, especially after surgery. But we can’t, we don’t have that extra time because we have to gown up because something else is going on. They’re just sort of sitting there and they’re like, “Well what am I even doing here, like, you’re not even – you’re not with me” as much as they would like right or as much as we would like. [Site 2 025]  So with that we were definitely quite pushed back in our ability to mobilize patients early. And so because of that the outcome of that is for every day that somebody is in bed without moving it equals about a week of constant exercise of rehab. And so if you imagine piling up those days back to back, let’s say somebody’s in bed for four to five days because of COVID and how ill they are, for an elderly person that translates to about an additional, like, four weeks in hospital, for example. [Site 3 001]  Unfortunately for my patients when I was redeployed especially, they probably suffered a little bit, you know, my colleagues were covering, but their first duty was to their own patients. So my group of patients who were getting diagnosed or I had been following weren’t getting the care they should have been receiving at the time. [Site 3 011]  I struggled was when I had to use an iPad to connect patients to family members and it could be in a very vulnerable situation, like a patient was dying, he doesn’t speak, English the daughter’s on the iPad, she’s crying, she can’t hold her dad, can’t hold his hand. You know, she’s trying to communicate with him; he’s extremely delirious. And that touch, that level of presence, from one of the most important people in his lives is missing and I’m the middle person. That was very awkward. And then the other part that I found very awkward to navigate was when people had spiritual care needs that could not be met through our spiritual care members because they are of external faith and they could not have those wishes met. And so there was a lot of anger or frustration from the family’s part when those aspects of care could not be fulfilled because of the restriction placed on our hospitals. [Site 3 012]  For instance, like you're giving medication and if you have time you can discuss every medication with the patient so they're educated and you're making sure you're giving them the right medication, they know why. And you're also reviewing it yourself. But if you don't have time, if you have another sick patient, you're just handing them the medication. Or going over very briefly, you know, it's not as careful, it's not as thorough, I guess. [Site 3 014]  At the beginning the Emergency Room was in a contingency state, which means there are way too many patients and there weren’t enough staff. So everything was just, “Do the best that you can through this.” For example, you’re supposed to do vital signs every four hours – but because they were so understaffed and there were so many patients through the COVID and the peak of the flu and stuff – you just do the best that you can. [SH029] |
